# Exogenous bacterial DnaK increases protein kinases activity in human cancer cell lines

**DOI:** 10.1186/s12967-021-02734-4

**Published:** 2021-02-09

**Authors:** Francesca Benedetti, Sabrina Curreli, Robert C. Gallo, Davide Zella

**Affiliations:** 1 Institute of Human Virology and Global Virus Network Center, Department of Biochemistry and Molecular Biology, University of Maryland School of Medicine, Baltimore, MD 21201 USA; 2 Institute of Human Virology and Global Virus Network Center, Department of Medicine, University of Maryland School of Medicine, Baltimore, MD 21201 USA

**Keywords:** Mycoplasma, DnaK, Kinase, Phosphorylation, Cancer

## Abstract

**Background:**

Studies of molecular mechanisms underlying tumor cell signaling highlighted a critical role for kinases in carcinogenesis and cancer progression. To this regard, protein kinases regulates a number of critical cellular pathways by adding phosphate groups to specific substrates. For this reason, their involvement in the complex interactions between the human microbiota and cancer cells to determine therapy and tumor progression outcome is becoming increasingly relevant. Mycoplasmas are components of the normal human microbiota, and several species have also been associated to human diseases, including certain cancers. It is also important to note that Mycoplasmas and their proteins are a component of the common tumor microenvironment. In addition, several epidemiological, in vivo and in vitro studies indicate a close involvement of Mycoplasmas in cellular transformation and cancer progression.

**Methods:**

In this study, we investigate the effect of exogenous Mycoplasma DnaK on kinases activity by treating in vitro four different eukaryotic cancer cell lines, namely lung and prostate cancer, colon adenocarcinoma, and neuroblastoma. Phosphorylation of kinases and specific substrates was measured at 20 and 60 min.

**Results:**

Kinome analysis of our data indicates that Mycoplasma DnaK promotes the dysregulation of the activity of specific kinases and their substrates, with a known involvement in carcinogenesis and cancer progression.

**Conclusions:**

Given the similarity in structure and amino acid composition of this protein with other bacterial DnaKs we provide a novel mechanism whereby components of the human microbiota and present in the tumor microenvironment are able to deregulate phosphorylation events occurring during carcinogenesis and cancer progression.

## Background

Protein kinases are key regulators of several cellular functions and by adding phosphate groups they can modify the activity of a protein, increase or decrease enzyme activity, direct the localization of many proteins, alter other biological activities such as transcription and translation. Moreover, some phosphorylation sites on a given protein are stimulatory while others are inhibitory [1]. The human genome encodes more than 500 protein kinases that transfer a γ-phosphate group from ATP to serine, threonine, or tyrosine residues [2, 3]. There are seven major groups of protein kinases, AGC (containing PKA, PKG, PKC families) [4], CAMK (Calcium/calmodulin-dependent protein kinase) [5], CK1 (Casein kinase 1) [6], CMGC (containing CDK, MAPK, GSK3, CLK families) [7], STE (Homologs of yeast Sterile 7, Sterile 11, Sterile 20 kinases) [8], TKL (Tyrosine kinase-like) [9], TK (Tyrosine kinase) [10] that can be further subdivided into families and sub-families based on sequence similarity and biochemical function [9]. A further eighth atypical kinases group assembles all the kinases that have no sequence similarity to typical kinases, but are known or predicted to have enzymatic activity, and a similar structural fold to typical kinases [11].

Recent advances in the understanding of the molecular mechanisms underlying tumor cell signaling have elucidated a critical role for kinases in carcinogenesis, from cellular transformation to promotion of metastasis [12]. Most protein kinases are involved in the processes of cell proliferation, survival and migration, and their dysregulation or overexpression mostly leads to hyper-phosphorylation of the target substrates. This dysregulation is implicated in several steps of cancer initiation and progression, as well as cancer recurrence [13–18]. Deregulation of kinase function may lead also to immunological, neurological and metabolic diseases [19]. Recently, several small-molecule kinase inhibitors have been developed for the treatment of diverse types of cancer where kinases hyperactivation is implicated in tumor progression, and many of these molecules have proven to be successfully in clinical therapy [12, 20]. Despite progress in tumor treatments, heterogenous cell population survive leading to tumor resistance. So far, the responsible mechanisms are only partially understood and they include pro-angiogenic signaling pathways activation [21] and multidrug resistance and antiapoptotic proteins upregulation [22–24]. However, most of the molecular mechanisms responsible for tumor dedifferentiation, aggressiveness and relapse related to kinases activation remain to be discovered.

Mycoplasmas are the smallest and simplest self-replicating bacteria belonging to the family of Mollicutes. They are part of the normal human microbiota, but several species have been associated to human diseases, like acute respiratory illness, genitourinary tract infections, joint infections and neurologic disorders [25–30]. They have also been associated with certain cancers, though the correlation is still unclear. However, several epidemiological [31], in vivo and in vitro studies indicate a close involvement of Mycoplasmas in cellular transformation and cancer progression [32–41].

Our group has previously demonstrated that a mycoplasmal protein, notably a chaperone protein belonging to the Heath shock protein (Hsp)-70 family, DnaK, binds Poly-(ADP-ribose) Polymerase (PARP)-1, a protein that plays a critical role in the pathways involved in DNA damage and repair by reducing its catalytic activity. It also binds USP10, a key p53 regulator, reducing p53 stability and its anti-cancer functions. We also observed tumorigenesis in vivo when Severe Combined Immune Deficient (SCID) mice were injected with Mycoplasma, and based on these results we proposed that Mycoplasma DnaK may have oncogenic activity through the inhibition of DNA repair and p53 functions [39, 40]. In addition, it has been recognized that DnaK belongs to a class of bacterial proteins that is also expressed on the surface of bacteria and secreted [42–45]. By interacting with receptors on the surface of cellular membranes and triggering their responses, these proteins exert a different function with respect to their original one and likely primary function. This “multitasking” capacity is called “protein moonlighting” and is becoming increasingly important to our understanding of mechanisms of bacterial pathogenicity associated with bacteria [46, 47].

In this study, we further investigate the ability of extracellular Mycoplasma DnaK to upregulate the activity of cellular kinases. We treated with a recombinant DnaK in vitro four different cell lines, representative of lung, prostate, colon adenocarcinoma, and neuroblastoma. We then analyzed the protein phosphorylation of kinases and their specific substrates at 20 and 60 min. Kinome analysis of our data indicates a selective dysregulation of the activity of certain kinases and their substrates involved in carcinogenesis and cancer progression. Our data indicate that Mycoplasma DnaK activates certain kinases known to be involved in different steps of tumorigenesis. Since some other bacterial DnaKs are similar in structure and amino acid composition, we provide a novel mechanism whereby components of the human microbiota are able to modify the activity of protein kinases implicated in carcinogenesis and cancer progression.

## Materials and methods

### Cell lines

A human colorectal carcinoma cell line (HCT116), an adenocarcinomic human alveolar basal epithelial cells (A549), a human prostate cancer cell line (PC-3), and a human neuroblastoma cell line (SH-SY5Y) used in the experiments were all from ATCC. The cells were cultured in a humidified incubator at 37 °C in 5% CO_2_ in McCoy medium (HCT116), F-12 K medium (A549 and PC-3), and F12 + EMEM (1:1) medium (SH-SY5Y), all containing 10% fetal bovine serum (FBS), 100 U/ml penicillin and 100 U/ml streptomycin. SH-SY5Y were also differentiated in neuron-like cells by adding Retinoic Acid (RA) (10 µM) to the culture for 7 days [48]. Briefly, cells were seed in T25 (or T75) flasks at a concentration of about 10^6^ cells/ml, maintained in an incubator at 37 °C, 5% CO_2_, and RA was added at the beginning of the differentiation process. The medium was replaced every 2–3 days, with concomitant addition of RA. During this period of time the cells were observed under direct light microscope to verify the progress toward differentiation into an elongated neuronal-like phenotype, as evidenced by a decreased amount in cell body clumping, and extension of numerous thin, branched neuritic processes that often connect to neighboring cells. The differentiated cells were finally used to analyze the effect of DnaK on kinases activation as described below.

### Expression and purification of *Mycoplasma fermentans* MF-I1 DnaK

Recombinant DnaK-V5 was obtained as previously described [39]. Briefly, MF-I1 DnaK sequence was inserted into a cloning vector, followed by the transformation and expression of the protein, subculture into TB/LB with Kanamycin, and subsequently fractionated and purified (Biomatik USA, Wilmington, DE). After purification, the protein was extensively dialyzed against PBS 1X, pH 7.4. Coomassie blue-stained SDS-PAGE (> 85%) was used to determine purity. Aliquots of the protein were kept at − 80 °C after reconstitution. Particular care was taken to avoid frequent freeze-thaws.

### Human phospho-kinase antibody array

Cells were treated with the recombinant DnaK-V5 protein (10 µg/ml) for 20 and 60 min, then harvested and lysed using the Lysis Buffer 6 from the Proteome Profiler Human Phospho-Kinase Array Kit (ARY003B, R&D Systems, Inc. USA & Canada). The amount of extracted proteins was measured using the Bradford assay (Bio-Rad). The Proteome Profiler Human Phospho-Kinase Array Kit was used to detect the relative levels of protein phosphorylation according to the manufacturer’s instruction. The signal produced is proportional to the amount of phosphorylation in the bound analyte. The spot signals were quantified using ImageJ software and normalized to the internal reference spots first and then to the corresponding not treated samples.

### Data analysis

A signal increase of 30% above the negative control (corresponding to 3 times the common 10% standard error in the assay) was considered a relevant effect in our analysis. In addition, activation of kinases between 20 and 30% was considered as a potential deregulation. KinMap, a web-based tool, allowed us to determine the association score (built in the application) of the activated kinases with cancer [49].

### Data visualization (Circos plots)

Circular visualization (Circos plot) of the overlapping phosphorylated kinases and substrates in the different cancer cell lines was performed using the software package for visualizing data and information, according to the distributor’s instructions (http://circos.ca/).

## Results

Deregulation of kinases activation is an established hallmark of cancer initiation and progression and several kinases and their downstream pathways have recently become important targets for the treatment of several cancers [50]. In order to verify if purified and exogenously added Mycoplasma DnaK has a role in kinases activation triggered by membrane cellular receptors, we treated different cancer cells lines in vitro with the *Mycoplasma fermentans* DnaK. Cells were harvested at early time points following stimulation (20 and 50 min). We reasoned that this short time points were appropriate to observe both early transduction signals and more immediate downstream events involving typical target substrates for phosphorylation pathways. For this analysis we used an assay where antibodies specific for a number of phosphorylates proteins are deposited on a membrane. Following stimulation with DnaK for the indicated period, the cell lysates are incubated with the membrane and processed following the manufacturer’s instructions. By binding the phosphorylated protein to the specific antibody, each spot allows for detection, quantification and direct comparison of the amount of phosphorylated proteins, including both kinases and the most relevant target substrates. The latter were important indicators of the downstream events triggered by the kinase activation.

**Fig. 1:**
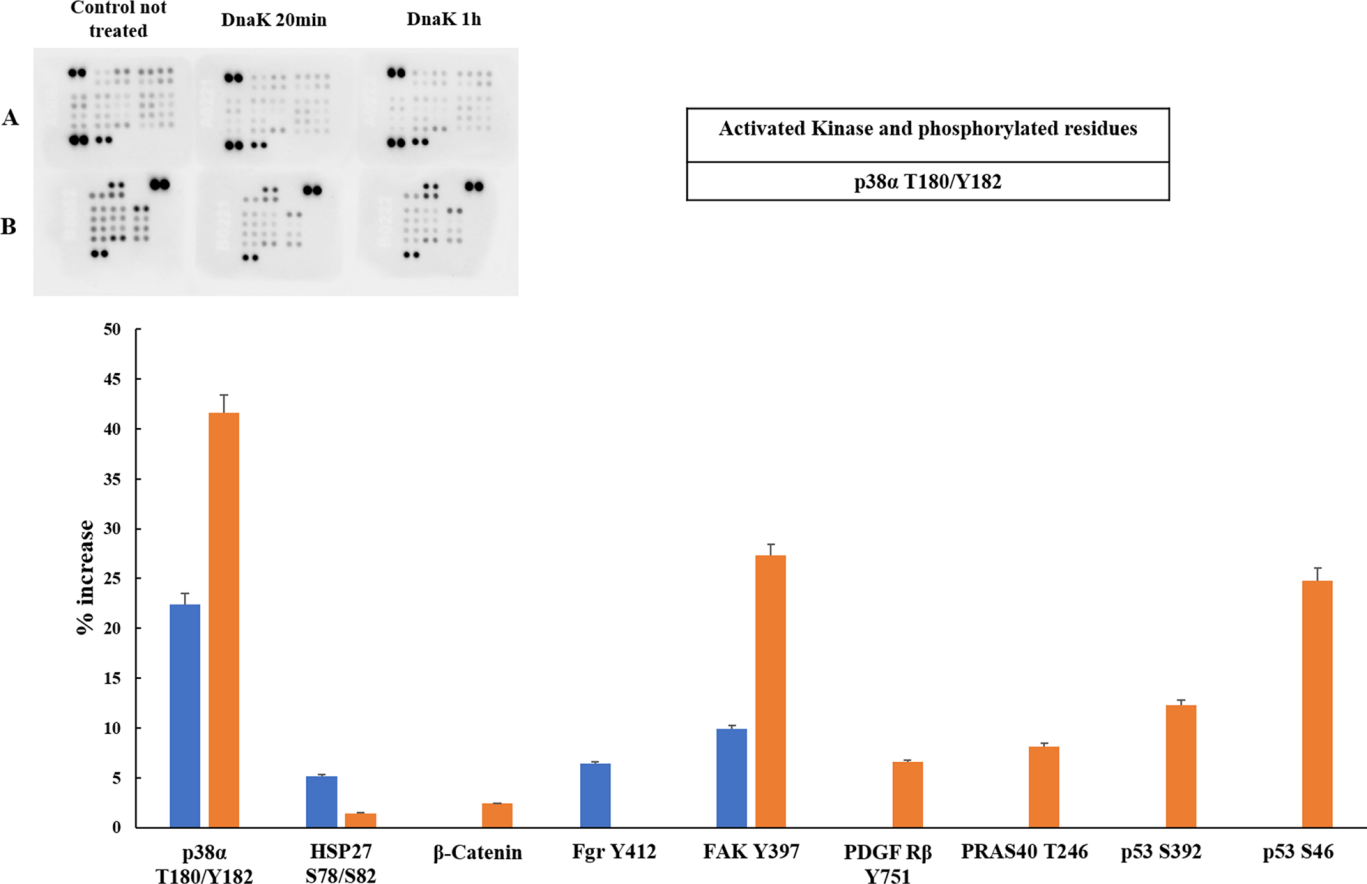
Human Phospho-Kinase Arrays for A549 cells and relative percentage increase of protein phosphorylation after 20 and 60 min of incubation with the exogenous and purified DnaK. The signal produced is proportional to the amount phosphorylation in the bound analyte. The spot signals have been normalized to the internal reference spots first and then to the corresponding not treated sample. The table lists the activated kinases with the relatives phosphorylated residues with signal increase above 30% of the negative control. In blue: activated Kinase and phosphorylated residues after 20 min of incubation with DnaK; in orange: activated Kinase and phosphorylated residues after 60 min of incubation with DnaK

**Fig. 2:**
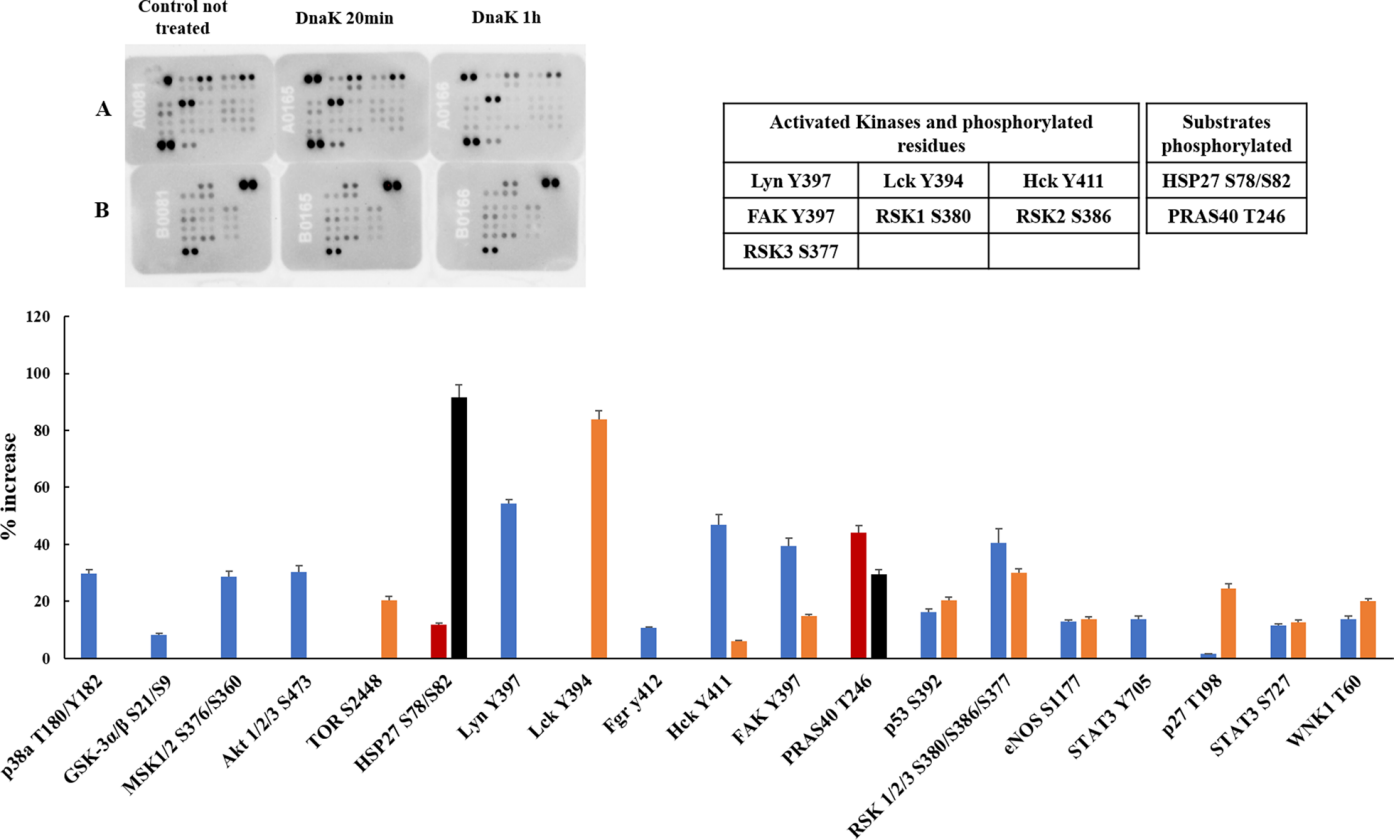
Human Phospho-Kinase Arrays for HCT116 cells and relative percentage increase of protein phosphorylation after 20 and 60 min of incubation with the exogenous and purified DnaK. The signal produced is proportional to the amount phosphorylation in the bound analyte. The spot signals have been normalized to the internal reference spots first and then to the corresponding not treated sample. The table lists the activated kinases and substrates with the relatives phosphorylated residues with signal increase above 30% of the negative control. In blue: activated Kinase and phosphorylated residues after 20 min of incubation with DnaK; in orange: activated Kinase and phosphorylated residues after 60 min of incubation with DnaK; in red: substrates phosphorylated after 20 min of incubation with DnaK; in black: substrates phosphorylated after 60 min of incubation with DnaK

**Fig. 3:**
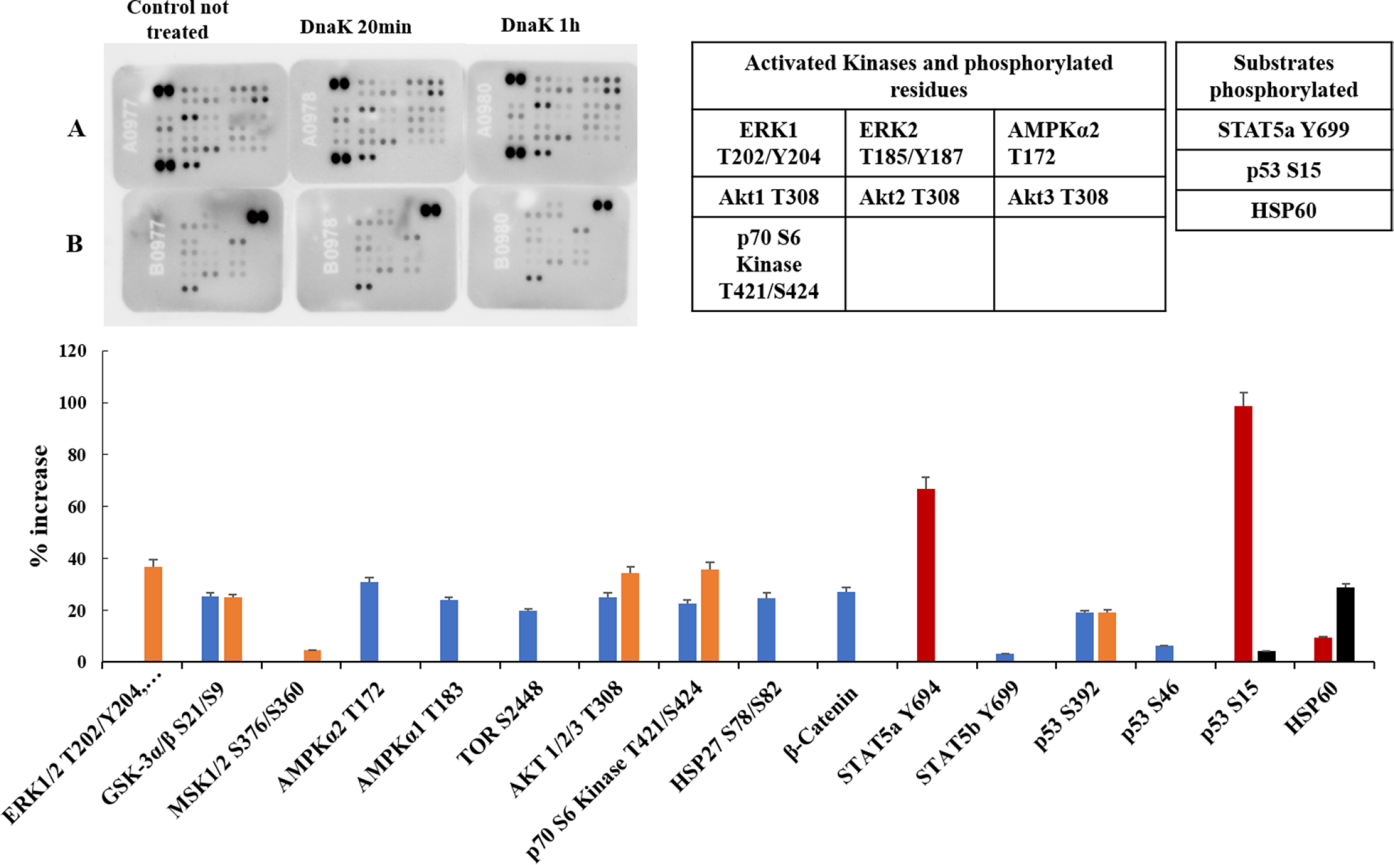
Human Phospho-Kinase Arrays for PC-3 and relative percentage increase of protein phosphorylation after 20 and 60 min of incubation with the exogenous and purified DnaK. The signal produced is proportional to the amount phosphorylation in the bound analyte. The spot signals have been normalized to the internal reference spots first and then to the corresponding not treated sample. The table lists the activated kinases and substrates with the relatives phosphorylated residues with signal increase above 30% of the negative control. In blue: activated Kinase and phosphorylated residues after 20 min of incubation with DnaK; in orange: activated Kinase and phosphorylated residues after 60 min of incubation with DnaK; in red: substrates phosphorylated after 20 min of incubation with DnaK; in black: substrates phosphorylated after 60 min of incubation with DnaK

**Fig. 4:**
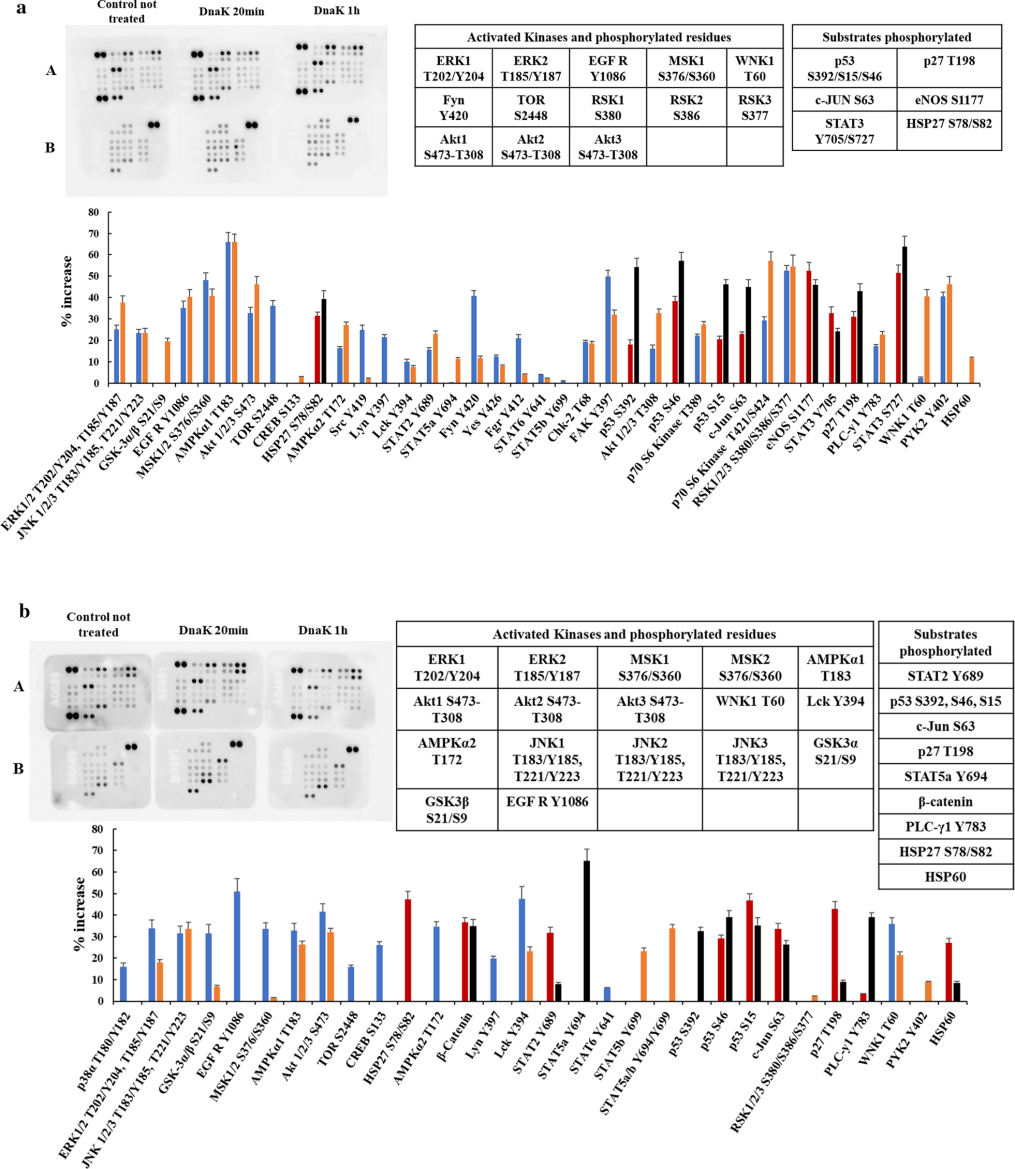
Human Phospho-Kinase Arrays for SH-SY5Y not differentiated cells (**a**) and SH-SY5Y differentiated cells (**b**) and relative percentage increase of protein phosphorylation after 20 and 60 min of incubation with the exogenous and purified DnaK. The signal produced is proportional to the amount phosphorylation in the bound analyte. The spot signals have been normalized to the internal reference spots first and then to the corresponding not treated sample. The table lists the activated kinases and substrates with the relatives phosphorylated residues with signal increase above 30% of the negative control. In blue: activated Kinase and phosphorylated residues after 20 min of incubation with DnaK; in orange: activated Kinase and phosphorylated residues after 60 min of incubation with DnaK; in red: substrates phosphorylated after 20 min of incubation with DnaK; in black: substrates phosphorylated after 60 min of incubation with DnaK

We detected a different kinase response among the cell lines analyzed, in that one of them responded very poorly (A549), one was stimulated to a very high degree (SH-SY5Y), while HCT116 and PC-3 fell somewhere in between (compare Figs. 1, 2, 3, 4).

### Effect of DnaK on A549 lung adenocarcinoma cell line

More in detail, we note that only one kinase, namely p38, was stimulated in A549 cell line at 20 min and its activation increased at a longer time point, about 1 h, while no substrates were phosphorylated above our threshold level. Slightly below our threshold level, one additional kinase was activated (FAK) and p53 was phosphorylated in position S46 (Fig. 1 and Table 1). Phosphorylation of p53 at Ser46 upregulates the ability of p53 to induce apoptosis [51].

**Table 1:** List of phosphorylated kinases in cell lines treated with exogenous, purified DnaK

Phosphorylated kinase	Cell line					Kinase family	Cancer association score
	A549	HCT116	PC3	SHSY5Y not differentiated	SHSY5Y differentiated		
EGF R Y1086				x	x	TK	1
FAK Y397		x		x		TK	1
Fgr Y412						TK	1
Fyn Y420				x		TK	1
Hck Y411		x				TK	1
Lyn Y397		x				TK	1
Lck Y394		x			x	TK	1
PDGF Rβ Y751						TK	1
PYK2 Y402						TK	0.263
Src Y419						TK	1
Yes Y426						TK	1
Akt1/2/3 S473-T308			x	x	x	AGC	1
MSK1/2 S376/S360				x	x	AGC	N/A
p70 S6 Kinase T421/S424			x			AGC	0.594
RSK1/2/3 S380/S386/S377		x		x		AGC	0.490/0.516/0.872
AMPKα1 T183					x	CAMK	0.608
AMPKα2 T172			x		x	CAMK	0.983
Chk-2 T68						CAMK	1
ERK1/2/3 T202/Y204, T185/Y187			x	x	x	CMGC	0.718/0.785
GSK3α/β S21/S9					x	CMGC	1
JNK1/2/3 T183/Y185, T221/Y223					x	CMGC	0.572/0.516/0.440
p38a T180/Y182	x					CMGC	0.291
WNK1 T60				x	x	STE	0.283
TOR S2448				x		Atypical	N/A

Numbers represents phosphorylation sites

*S* serine, *T* threonine, *Y* tyrosine, *x* protein phosphorylated

### Effect of DnaK on HCT116 colorectal carcinoma cell line

In the HCT116 cell line, exogenous DnaK stimulated multiple phosphorylation events involving several kinases belonging to the TK family (namely FAK, Hck, Lyn and Lck) and the RSK1/2/3 group (of the AGC family) increased their phosphorylation status by more than 30%, between 20 and 50 min of treatment. A number of other kinases were also activated just slightly below the threshold (namely p38a, MSK1/2, Akt1/2/3, WNK1 and p27). Two substrates were phosphorylated, namely HSP27 (above the threshold) and PRAS40 (just below threshold) (Fig. 2, Tables 1 and 2). HSP27 is a member of the small Hsp family, and it is one of the major players of many signaling pathways leading to carcinogenesis, resistance to anti-cancer-drug treatment, and apoptosis inhibition. Several studies report that HSP27 is overexpressed in many types of cancer and its functions are mainly regulated by phosphorylation [52]. PRAS40 is a substrate of Akt, with several effects on cell metabolism including cell survival and growth [53], and it has been implicated in different pathologic conditions, including cancer and insulin resistance [54–58].

**Table 2:** List of phosphorylated substrates in cell lines treated with exogenous, purified DnaK

Phosphorylated substrate	Cell line			
	HCT116	PC3	SHSY5Y not differentiated	SHSY5Y differentiated
β-Catenin				x
CREB1				
HSP27	S78/S82		S78, S82	S78, S82
HSP60	x			x
c-JUN			S63	S63
eNOS			S1177	
p27			T198	T198
p53		S15	S15, S46, S392	S15, S46, S392
PLC-γ1				Y783
PRAS40	T246			
STAT2				Y689
STAT3			Y705, S727	
STAT5a		xY699		xY694

Numbers represents phosphorylation sites

*S* serine, *T* threonine, *Y* tyrosine, *P* protein phosphorylated, unknown site

### Effect of DnaK on PC-3 prostate cancer cell line

The kinases activated above the threshold by DnaK in PC-3 cell line were the Akt1/2/3 group and p70 S6 kinase of the AGC family, AMPKα2 of the CAMK family, and the ERK1/2/3 group of the CMGC family. A number of others were activated just below threshold (namely GSK-3α/β, AMPKα1, TOR, HSP27 and β-catenin. Three substrates were phosphorylated, namely STAT5α, p53 in Ser 15 and HSP60 (Fig. 3, Tables 1 and 2). Once phosphorylated, STATs forms homo- or heterodimers that translocate to the nucleus and function as transcription factors. Dysregulation of STAT5α has been observed in cellular invasion, angiogenesis and immune evasion, and inhibition of STAT5α has been demonstrated to enhance the sensitivity to cisplatin and 5FU in vitro [59]. P53 is arguably the most important anti-cancer protein in that, upon DNA damage, controls the correct and synchronized execution of both cell cycle arrest and apoptosis to allow for proper DNA repair. Phosphorylation of one of the key residues, serine 15, has been shown to coordinate polyphosphorylation of p53 [60], and stabilize the protein through disruption of MDM2 binding [61]. Phosphorylation of serine 15 also prevents p53 from being exported from the nucleus and stimulates p53 transcriptional activity through the increased association with p300 coactivator [62]. For this reason, dysregulation of p53 can have profound effect on a cell’s fate. HSP60 is a mitochondrial chaperon protein involved in protein import and assembly of macromolecules, which can also be present in the cytosol. Some studies have suggested that it has anti-apoptotic effect by inhibiting caspase-3 activity [63, 64]. Of note, the phosphorylation pattern of PC-3 cells was completely different that the one observed in HCT116.

### Effect of DnaK on SH-SY5Y neuroblastoma cell line

**Fig. 5:**
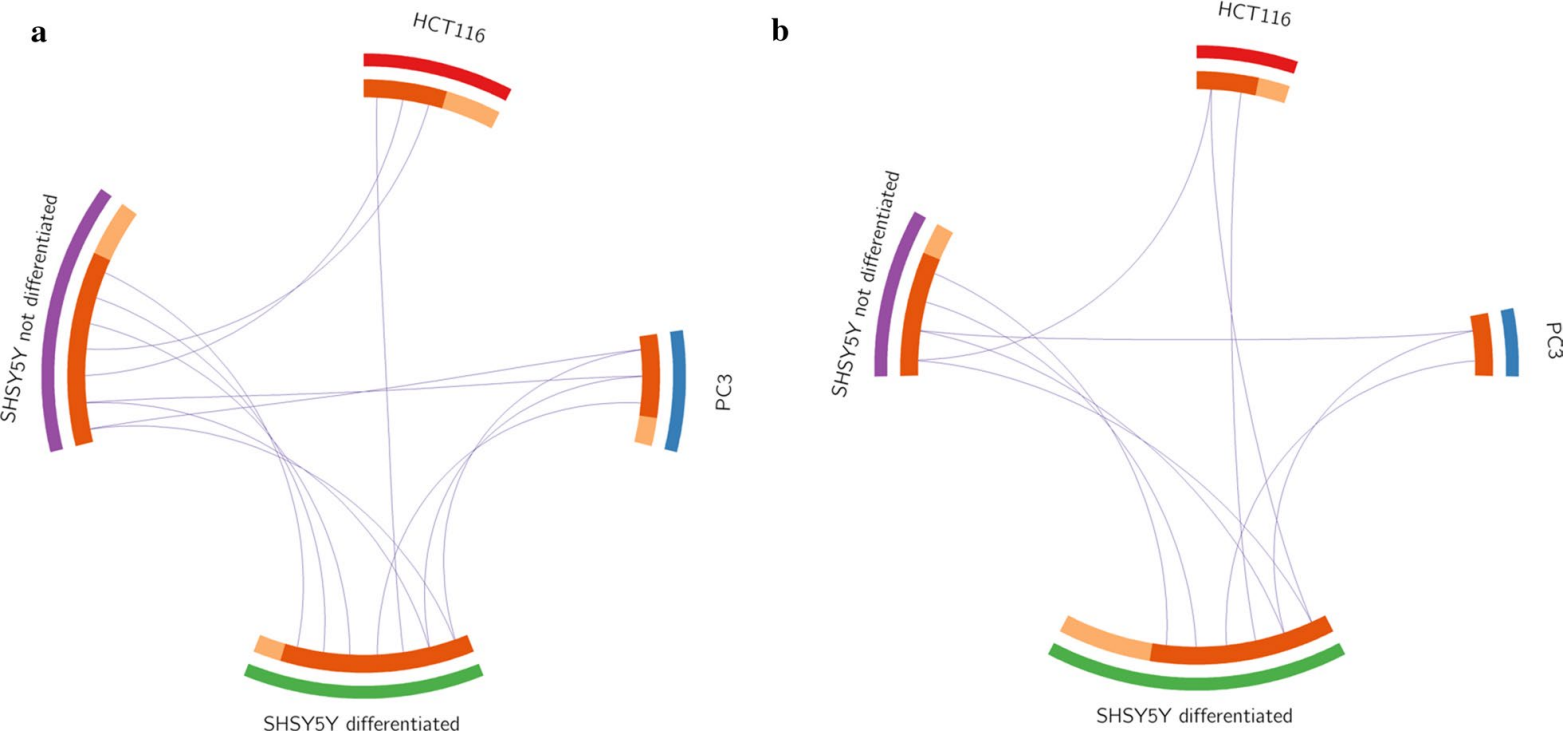
Circos plot of the phosphorylated substrates (**a**) and of the phosphorylated kinases (**b**). The Circos plots show how phosphorylated substrates (**a**) and phosphorylated kinases (**b**) in the different cell lines overlap. On the outside, each arc represents the identity of each cell line. On the inside, each arc represents a phosphorylated substrate, where each substrate has a spot on the arc (cell line). Dark orange color represents the phosphorylated substrates that appear in multiple cell lines and light orange color represents phosphorylated substrates that are unique to that cell type. Purple lines link the same phosphorylated substrate that is shared by multiple cell lines. The greater the number of purple links and the longer the dark orange arcs implies greater overlap among the phosphorylated substrate

A dynamic picture emerged by comparing SH-SY5Y cells differentiated and not differentiated. In fact, while they both showed widespread kinases phosphorylation, some kinases remained active through the differentiation phase while the activity of other decreased and new ones were activated in their place (compare, Fig. 4a, b and Table 1). More in detail, EGF R, Akt 1/2/3 group, MSK1/2, ERK1/2/3 group and WNK1 were active in both differentiated and undifferentiated stages (Table 1). Fyn, FAK and TOR were active only in the not differentiated cells, with an additional number of kinases active just below the threshold level (JNK 1/2/3, AMK1α1, Src, Lyn, STAT2, Fgr, p70 S6 kinase, p27) (Fig. 4a and Table 1). In the differentiated stage Lck, AMPKα1/2, JNK 1/2/3 group and GSK-3α/β were active above threshold, while CREB, STAT5β, PYK2 were active slightly below threshold (Fig. 4b and Table 1). Two substrates were phosphorylated in both stages (HSP27, c-Jun and p27) (Table 2), two were phosphorylated only in not differentiated cells (STAT3 and eNOS) (Fig. 4a and Table 2), and five more were phosphorylated in differentiated cells (STAT5α, STAT2, β-catenin, HSP60, PLC-γ1) (Fig. 4b and Table 2). Phosphorylated HSP27 increases cell invasion, enhances cell proliferation and suppresses FAS-induced apoptosis in vitro [65]. Phosphorylated c-Jun dimerizes with c-Fos to form the transcription factor AP-1, and it is involved in cell cycle progression and apoptosis [66]. eNOS generates intracellular Nitric Oxide, which is involved in many cancer-related events [67]. p27 is an inhibitor of cyclin D dependent kinases, and is involved both in cell proliferation and differentiation [68]. PLC-γ1 (phospholipase gamma 1) is mostly involved in the intracellular transduction of receptor-mediated tyrosine kinase signaling cascade [69]. STAT2 dysregulation may lead to activation of the pro-oncogenic STAT3 pathway [70]. These data are indeed better evaluated when the overlapping phosphorylated kinases and substrates in the different cancer cell lines were visualized using the Circos plot software (see “Materials and methods”) (Fig. 5a, b). Overall, it seems that differentiation of SH-SY5Y is associated to a “switch” in the activation of certain kinases. In fact, FAK, Fyn and RSK1/2/3 change from phosphorylated in not differentiated cells, to non-phosphorylated in differentiated cells, while Lck, AMPKα1/2, GSK3α/β and JNK1/2/3 change from not phosphorylated in not differentiated cells, to phosphorylated in differentiated cells (Table 1). In addition, we also observed a broadening of the number of phosphorylation substrates, with the inclusion of β-catenin, HSP60, PLC-γ1, STAT2 and STAT5a in differentiated cells, while eNOS and STAT3 were not phosphorylated (Table 2 and Fig. 5a, b).

## Discussion

Kinases play a prominent role in signal transduction and co-ordination of several complex cellular functions. In particular, their involvement in cellular proliferation and differentiation makes them important players and targets during the different steps of tumorigenesis [12]. We proposed Mycoplasma DnaK, a bacterial chaperone, as a potential link between the association of certain Mycoplasma with cancers and the molecular mechanisms involved in cellular transformation [39, 40]. Moreover, the ability of DnaK to be expressed on the bacterial surface and to bind other protein of the cellular membrane makes it a good candidate for further studies aimed at understanding its ability to influence tumor progression [42, 44, 46, 47].

To our knowledge, this is the first time that an exogenous bacterial DnaK has been studied in the context of interaction with human receptor-mediated kinases present on different cancer cells and their subsequent activation. In this paper, we investigated the possibility that exogenous and purified DnaK added to cancer cell lines could activate specific kinases involved in carcinogenesis and cancer progression. Our experimental settings relied on an in vitro assay to measure kinase activation at different time point, and on the Kinome activity pathway analysis. We selected different cell lines representative of different cancer types, namely colon adenocarcinoma, lung and prostate cancer [36, 71]. It is important to note that Mycoplasmas and their proteins are a component of the common tumor microenvironment surrounding all the three tissues [72]. The fourth cancer line, a neuroblastoma cell line, was selected because of the increasingly important association between gut and brain [73, 74], and the hypothesis that microbial proteins released by the gut microbiota (to which Mycoplasmas belongs) could influence brain functions and cancers [75].

Our data clearly show that exogenous DnaK is able to trigger activation of certain kinase-related transduction pathways in a cell-specific way. In particular, while lung carcinoma seems to be the least sensitive, a notable effect was seen on both undifferentiated neuroblastoma cells, and more so in the differentiated ones. The effect on colon carcinoma and prostate cancer cell lines was in the middle of the range observed in the neuroblastoma cells. The reason for this apparent cell-specific response is not clear. The most likely hypothesis is that differentiation either cause selective expression of certain cellular receptors on particular cancer cells, able to bind exogenous DnaK and transduce of kinase cascade [76–78]. In addition, differentiation could also increase expression of specific kinases, and indeed differentiated neuronal cells have increased expression of FAK, essential for cell adhesion and migration [79]. Kinases activation in turn caused the phosphorylation of specific substrates, which would indicate engagement of certain particular cell functions. Among the different kinases activated in our in vitro system, a substantial number are found strongly associate with certain cancers, as indicated by the association score. Indeed, most of the kinases phosphorylated in the presence of DnaK play a role in the DNA double strand response (both sensing and recruitment of other proteins for repair), cell cycle and apoptosis, metabolism, cell differentiation and proliferation.

It is important to note that the DnaK-mediated kinase activation profile of one cell line belonging to a particular cancer type may be cell-line specific and not necessarily reflect the DnaK-mediated kinase activation signature of this cancer type. The analysis of multiple cell lines of a particular cancer type would be needed to establish such a signature.

To better study the role of exogenous DnaK and expand our results by including additional cell types, it should be of interest to analyze DnaK-mediated kinase activation of micro-environmental cells that have well characterized roles in cancer progression such as Tumor-Associated Macrophages (TAMs) or Cancer-Associated Fibroblasts (CAFs). Additional experiments are also needed to characterize the effects that may result from the stimulation of these kinases on different cellular pathways and their outcome(s) on the cellular differentiation ability and functions. Finally, we point out that one limitation of our assay consists in the lack of properly address the response of other important kinases not included in the array, like for example the PKC family whose member are involved in regulating important cell functions [80, 81], and for this reason further experiments are also needed to better understand the potential relationship between DnaK and other potentially relevant kinases.

Based on our data, we hypothesize that the presence of exogenous DnaK in the tumor microenvironment may contribute to a status of cellular hyperactivation. In fact, even if our in vitro settings are very different from the situation in vivo, nonetheless the complex tumor microenvironment with its milieu of pro-inflammatory cytokines is most likely stimulating cancer cells to express a number of receptors, potentially providing additional binding targets for DnaK which then would trigger activation of specific kinases. In turn, this could result in dysregulated cellular pathways, including reduced repair activity, increased proliferation and reduced response to pro-apoptotic signals of certain tumors (or tumor stages). Our data identify a potential role of Mycoplasma DnaK for cancer progression, and given the similarity in structure and aminoacid composition of this protein with other bacterial DnaKs, such as *H. pylori* and *F. nucleatum* [39] we provide a novel mechanism whereby components of the human microbiota are able to promote carcinogenesis and cancer progression once released in the tumor microenvironment [82]. Additional experiments are ongoing to identify the receptors able to bind to DnaK and transduce the signals.

## Conclusions

Our results point to a new role for Mycoplasma DnaK, and by extension other bacterial DnaKs with similar structure and aminoacidic compositions, present in the tumor microenvironment, in upregulating the phosphorylation activity of certain kinases involved in carcinogenesis and cancer progression. Our data could be relevant in determining a new role for this bacterial chaperon protein thus potentially establishing a new target for anti-cancer therapy aimed at reducing cancer progression.

## Data Availability

All data utilized, generated or analyzed during these studies are included in this published article.

## Abbreviations

p38α: P38 mitogen-activated protein kinases
ERK1/2: Extracellular signal-regulated kinases
JNK 1/2/3: C-Jun N-terminal kinases
Hck: Hematopoietic cell kinase
Lyn: Lck/Yes novel tyrosine kinase
Lck: Lymphocyte-specific protein tyrosine kinase
RSK1/2/3: 90 KDa ribosomal S6 kinase
MSK1/2: Mitogen and stress activated protein kinase
Akt1/2/3: Protein kinase B
p27: Cyclin-dependent kinase inhibitor 1B
HSP27: Heat shock protein 27
PRAS40: Proline-rich Akt substrate of 40 kDa
FAK: Focal Adhesion Kinase
GSK-3α/β: Glycogen synthase kinase 3
AMPK: AMP-activated protein kinase
TOR: Target of rapamycin
WNK1: Lysine deficient protein kinase 1
Fgr: Gardner-Rasheed feline sarcoma viral (v-fgr) oncogene homolog
EGF R: Epidermal growth factor receptor
CREB: CAMP response element-binding protein
Src: Proto-oncogene tyrosine-protein kinase Src
Fyn: Proto-oncogene tyrosine-protein kinase Fyn
Yes: Proto-oncogene tyrosine-protein kinase Yes
Chk-2: Checkpoint kinase 2
PLC-γ1: Phosphoinositide phospholipase C isoform γ
PYK2: Proline-rich tyrosine kinase 2
HSP60: Heat shock protein 60
PDGF Rβ: Platelet-derived growth factor receptor beta
p53: Tumor protein p53
eNOS: Endothelial NOS also known as nitric oxide synthase 3
STAT: Signal transducer and activator of transcription protein family
